# Early impairment of skeletal muscle endothelial glycocalyx barrier properties in diet‐induced obesity in mice

**DOI:** 10.1002/phy2.194

**Published:** 2014-01-06

**Authors:** Bart J. M. Eskens, Thomas M. Leurgans, Hans Vink, Jurgen W. G. E. VanTeeffelen

**Affiliations:** 1 Department of Physiology, Cardiovascular Research Institute Maastricht (CARIM), Maastricht University, Maastricht, The Netherlands

**Keywords:** Glycocalyx, high‐fat diet fed mouse model, insulin sensitivity, Sidestream Dark‐Field imaging

## Abstract

While previous studies have indicated an important role for the endothelial glycocalyx in regulation of microvascular function, it was recently shown that acute enzymatic glycocalyx degradation in rats was associated with an impaired insulin‐mediated glucose disposal. The aim of this study was to determine whether glycocalyx damage in skeletal muscle occurs at an early stage of diet‐induced obesity (DIO). The microcirculation of the hindlimb muscle of anesthetized C57Bl/6 mice, fed chow (CON) or a high‐fat diet (HFD) for 6 and 18 weeks (w), respectively, was visualized with a Sidestream Dark‐Field camera, and glycocalyx barrier properties were derived from the calculated perfused boundary region (PBR). Subsequently, an intraperitoneal glucose tolerance test was performed and the area under the curve (AUC) of blood glucose was calculated. Impairment of glycocalyx barrier properties was already apparent after 6 weeks of HFD and remained after 18 weeks of HFD (PBR [in *μ*m]: 0.81 ± 0.03 in CON_6w vs. 0.97 ± 0.04 in HFD_6w and 1.02 ± 0.07 in HFD_18w [both *P* < 0.05]). Glucose intolerance appeared to develop more slowly (AUC [in mmol/L × 120 min]: 989 ± 61 in CON_6w vs. 1204 ± 89 in HFD_6w [*P* = 0.11] and 1468 ± 84 in HFD_18w [*P* < 0.05]) than the impairment of glycocalyx barrier properties. The data indicate that damage to the endothelial glycocalyx is an early event in DIO. It is suggested that glycocalyx damage may contribute to the development of insulin resistance in obesity.

## Abstract (precis)

In this study we assessed glycocalyx barrier properties in skeletal muscle using Sidestream Dark‐Field imaging at an early and later stage of diet‐induced obesity in mice, by feeding them a high‐fat diet for 6 and 18 weeks, respectively. Glycocalyx barrier properties in hindlimb muscle microcirculation were found to be impaired after 6 weeks already. Our results suggest that in obesity glycocalyx damage represents an early aspect of microvascular dysfunction which may as well contribute to the development of glucose intolerance.

## Introduction

Obesity is a growing global health problem, as it has been associated with an increased risk for developing cardiovascular complications and type II diabetes. Endothelial dysfunction has been indicated to play a central role in both aspects (Schalkwijk and Stehouwer 2005); Kim et al. 2006). At the luminal side of the endothelium resides the endothelial glycocalyx, consisting of proteoglycans and glycosaminoglycans in dynamic association with the plasma and its soluble components (Pries et al. 2000); Tarbell and Pahakis 2006); Reitsma et al. 2007); Van Teeffelen et al. 2007). The glycocalyx contributes to the regulation of multiple aspects of endothelial function (Nieuwdorp et al. 2008), including the delivery and exchange of plasma solutes and hormones (VanTeeffelen et al. 2007); Woodcock and Woodcock 2012), shear‐mediated release of nitric oxide, and homeostasis of the vascular wall (Gouverneur et al. 2006); Lopez‐Quintero et al. 2009). Previous animal and human studies showed that severe acute (Vink et al. 2000); van den Berg et al. 2009) as well as long‐term hyperlipidemic (Constantinescu et al. 2011) and hyperglycemic conditions (Zuurbier et al. 2005); Nieuwdorp et al. 2006) resulted in significant reductions in whole‐body glycocalyx volume and impaired microvascular glycocalyx barrier properties (Constantinescu et al. 2003), 2011); Zuurbier et al. 2005); Broekhuizen et al. 2010). Consequently, the hypothesis has been put forward that glycocalyx loss may be an important step in the development of endothelial dysfunction during conditions of increased cardiovascular risk (Nieuwdorp et al. 2008); Noble et al. 2008). Recently, we provided evidence in rats that the endothelial glycocalyx also plays a role in the regulation of insulin sensitivity (Eskens et al. 2013). We showed in rats that insulin‐mediated increases in microvascular blood volume in muscle included an increased accessibility of circulating blood into the glycocalyx; the importance of this effect in the metabolic action of insulin was illustrated by the observation that enzymatic degradation of the glycocalyx using hyaluronidase was associated with an acute impairment of insulin‐mediated glucose disposal from the blood (Eskens et al. 2013). These recent data suggest that loss of glycocalyx may, thus, not only be a consequence of a disturbance in glucose metabolism (Zuurbier et al. 2005); Nieuwdorp et al. 2006), but may as well contribute to it, thereby providing a target which links endothelial dysfunction to insulin resistance. To appreciate this contribution better, it is important to know whether glycocalyx damage is a relatively early process in the development of insulin resistance during risk factor exposure. Therefore, we determined whether glycocalyx damage occurs at a relative early stage of diet‐induced obesity (DIO). In particular, we wanted to know whether glycocalyx damage would be manifested in the microcirculation of skeletal muscle, as this tissue constitutes the major site for insulin‐mediated glucose disposal in the body.

In this study we used the high‐fat diet (HFD)‐fed C57BL/6 mouse model, which is a widely used animal model to study mechanisms of impaired glucose tolerance and (early) type 2 diabetes (Winzell and Ahren 2004); Costa et al. 2011). Previous studies showed that feeding these mice with a HFD for several weeks induced visceral fat deposition and was associated with a progressive loss of insulin sensitivity over time (Winzell and Ahren 2004). Furthermore, HFD has been indicated to induce endothelial dysfunction in large vessels (Molnar et al. 2005); Ketonen et al. 2010). We assessed the occurrence of glycocalyx damage in skeletal muscle microcirculation at a relative early (6 weeks) and at a later (18 weeks) stage of DIO, and related this to the development of glucose intolerance in this model.

## Methods

### Animals and diet

The experimental protocols were approved by the Animal Ethics Care and Use committee of Maastricht University (AEC protocol numbers: 2010‐101). After arrival from the external supplier (Harlan, Horst, The Netherlands) 4–5 weeks old male mice (C57Bl/6; *n* = 31) were housed at the animal facility of Maastricht University and received standard chow (Ssniff GmbH, Soest, Germany, containing on caloric basis 9% fat, 58% carbohydrates, and 33% protein) for 6 weeks (CON_6w; *n* = 7) and 18 weeks (CON_18w; *n* = 8), or a HFD (Research Diets, New Brunswick, NJ, containing on caloric basis 60% fat, 20% carbohydrate, and 20% protein), also for 6 weeks (HFD_6w; *n* = 8) and 18 weeks (HFD_18w; *n* = 8), respectively. All animals had unrestricted access to water. Weekly measurements of body weight, blood pressure using a CODA non‐invasive blood pressure monitoring system (Kent Scientific, Torrington, CT), and glucose levels and plasma insulin levels via blood sampling of the saphenous vein were performed in the conscious animal after a morning fast (4 h). Blood glucose (~5 *μ*L) was measured with a glucose meter (Ascencia Contour, Bayer, Mijdrecht, the Netherlands), and about 40 *μ*L blood was collected from the tail using a 75‐*μ*L glass capillary tube (Hirschmann, Eberstadt, Germany) to measure plasma insulin levels with an ELISA (ALPCO Diagnostics, Salem, NH).

### Experimental protocol

At the day of experiment, after an overnight fast (10–12 h), mice were anesthetized using an intraperitoneal injection of 0.39 mg/kg fentanyl, 7.81 mg/kg midazolam, and 7.81 mg/kg acepromazine (Coomans et al. 2011). Anesthesia was maintained by an additional bolus after 60–90 min of 0.10 mg/kg fentanyl, 1.56 mg/kg midazolam, and 1.56 mg/kg acepromazine. The combination of fentanyl, midazolam, and acepromazine has been recently introduced in mice studies of insulin sensitivity (van den Berg et al. 2010); Coomans et al. 2011). The animal was put in a prone position and body temperature was monitored with a rectal probe and maintained at ~37°C with the use of a heating pad and lamp. During anesthesia a cannula was inserted intraperitoneally and the superficial muscles on the dorsal upper part of the hindlimb, that is, the biceps femoris and the semitendinosus muscles, were exposed by a small incision in the skin. The hindlimb muscles were suffused with a bicarbonate‐buffered physiological salt solution (PSS) of the following composition (in mmol/L): 131.9 NaCl, 4.7 KCl, 2.0 CaCl_2_, 1.2 MgSO_4_, 20 NaHCO_3_, and equilibrated with 5% CO_2_–95% N_2_ to obtain a pH of ±7.4. Preparations were equilibrated for 20 min and then glycocalyx barrier properties and glucose tolerance were measured.

#### Imaging of muscle microcirculation

To measure the effect of the HFD on glycocalyx barrier properties, the microcirculation of the hindlimb muscles was visualized five times at baseline for 10 min with a Sidestream Dark‐Field (SDF) camera (Eskens et al. 2013). The SDF camera is equipped with a 5× magnifying objective lens system‐containing probe, imaging the red blood cells (RBCs) in the tissue‐embedded microcirculation using green‐pulsed LED ring illumination (Goedhart et al. 2007). In two animals (one mouse fed chow for 18 weeks and one mouse fed a HFD for 18 weeks), imaging was not performed due to technical problems with the camera.

#### Intraperitoneal glucose tolerance test

To determine glucose tolerance, an intraperitoneal glucose tolerance test (IPGTT) was performed immediately after the SDF measurements. Mice were infused with a bolus of 1 g/kg glucose (0.1 g/mL) via the i.p. cannula. Blood glucose (~5 *μ*L) was measured, via tail bleeding, at *t* = −10 and 0 (pre) and *t* = 5, 10, 20, 30, 40, 50, 60, 70, 80, 90, 100, 110, and 120 min after the glucose infusion. In addition, about 40 *μ*L blood was collected from the tail using a 75 *μ*L glass capillary tube at *t* = 0 (pre) and *t* = 10, 30, 60, and 90 min after glucose infusion to determine systemic hematocrit and plasma insulin levels. Thus, in total, maximal of 275 *μ*L blood was collected in an animal during the IPGTT. It has been indicated that this amount does not cause local trauma (Diehl et al. 2001). In four animals (two control mice fed chow for 6 weeks and two mice fed chow for 18 weeks), glucose levels were only measured for up to 90 min after the glucose administration. Plasma insulin levels were not measured in five animals (two control mice fed chow for 6 weeks, one mouse fed HFD for 6 weeks, and two mice fed chow for 18 weeks) due to an insufficient amount of plasma collected.

#### Muscle capillary density

At the end of the IPGTT, when the animal was still under anesthesia, the hindlimb muscle was removed and immediately fixed in a 4% formaldehyde solution for subsequent histological analysis of capillary density. This measurement was performed in *n* = 4 mice fed chow for 6 weeks, in four mice fed a HFD for 6 weeks, in five mice fed chow for 18 weeks, and in five mice fed a HFD for 18 weeks.

### Data analysis

#### Measurements of glycocalyx barrier properties in muscle

The analysis of the glycocalyx barrier properties was performed blinded, and has been described previously (Vlahu et al. 2012); Eskens et al. 2013). Furthermore, the cartoon in Figure 2B depicts the analysis diagrammatically. Briefly, microvessels with a continuous RBC flow were manually selected in the recorded movies (100 frames). In each frame, lines were placed approximately every 10 *μ*m perpendicular to the vessel direction along the length of the microvessel. Each line represented a vessel segment; for each vessel segment a total of 21 parallel (every ±0.5 *μ*m) intensity profiles were plotted (using ImageJ, National Institutes of Health, Bethesda, MD) and RBC column width (full width half maximum) was determined for all 100 consecutive frames in a movie, revealing a total of 2100 RBC column width measurements for a vessel segment (21 profiles × 100 frames). The cumulative distribution of the RBC column widths for these 2100 measurements was constructed and used to determine median RBC column diameter (*D*_P50_); in addition, RBC column widths percentiles between P25 and P75 were fitted with a linear fit to determine the perfused diameter (*D*_perf_) for the vessel segment. The difference between the *D*_perf_ and the *D*_P50_ divided by two is defined as the perfused boundary region (PBR). The majority of the vessel segments had a median RBC column diameter between 4 and 6 *μ*m (Fig. 2C), and only segments of this size were considered in the analysis. All PBRs that were calculated within one individual recording were pooled. Subsequently, pooled PBRs were averaged for the five recordings that were made per experiment, resulting in a single PBR value per animal. The analysis is not expected to be affected by variations in blood volume because the derived PBRs were classified according to their diameter. Thus, for each vessel segment, the PBR was calculated per bin class of median RBC column width. An increase in blood volume is expected to be associated with an increase in the median RBC column, and as a result the calculated PBR will belong to a larger bin class.

#### Intraperitoneal glucose tolerance test

As a reflection of the circulating levels of glucose during the IPGTT we calculated the total area under the curve (AUC) of the glucose concentration versus time by the linear trapezoidal rule for the period of 0–120 min after glucose infusion (Fig. 3A); this was only done for those experiments in which blood was sampled for the entire 120 min (*n* = 5 for mice fed chow for 6 weeks, *n* = 7 for mice fed a HFD for 6 weeks, *n* = 7 for mice fed chow for 18 weeks, and *n* = 6 for mice fed a HFD for 18 weeks [*n* = 8]). As a reflection of the circulating insulin levels we calculated the AUC of the insulin concentration versus time by the linear trapezoidal rule for the period of 0–90 min after glucose infusion (Fig. 3B).

#### Muscle capillary density

Paraffin‐embedded hindlimb muscles were sectioned and from each muscle 3–6 slides were stained with 200 *μ*L FITC‐labeled lectin from triticum vulgaris (WGA‐FITC; 50 *μ*g/mL, Sigma Aldrich, Zwijndrecht, the Netherlands) for 30 min in the dark at room temperature (20°C), washed three times with phosphate buffered saline, and mounted with 4',6‐diamidino‐2‐phenylindole (Vectashield, Vector Laboratories, Southgate, U.K.). Tissues were visualized and photographed using a Leica DFC320 digital camera (Leica, Rijswijk, The Netherlands) at 400× magnification (Leica DM3000 microscope, Leica, Rijswijk, The Netherlands). System control and imaging processing were performed using Leica QWin Image Processing and Analysis morphometry software (Leica Microsystems, Cambridge, UK). For each slide capillary density was determined by counting the number of capillaries per mm² muscle surface area. For a muscle the values of all stained slides were averaged revealing 1 value of capillary density per animal.

### Statistical analysis

All data are presented as mean ± SEM. A two‐way analysis of variance (ANOVA) followed by post hoc Bonferroni correction was used for analysis of the weekly measurements of body weight, blood pressure, blood glucose, and plasma insulin levels measured in the conscious mice with diet and time as independent variables. Statistical differences between AUC of glucose levels, AUC of plasma insulin levels, and the PBR in the anesthetized mice were tested with Student's unpaired *t*‐tests. The group of mice that received chow for 6 weeks was used as reference group. Furthermore, the relationship between the PBR and the AUC of glucose (Fig. 4) was tested using correlation analysis. A value of *P* < 0.05 was considered statistically significant.

## Results

### Systemic data

**Figure 1.**
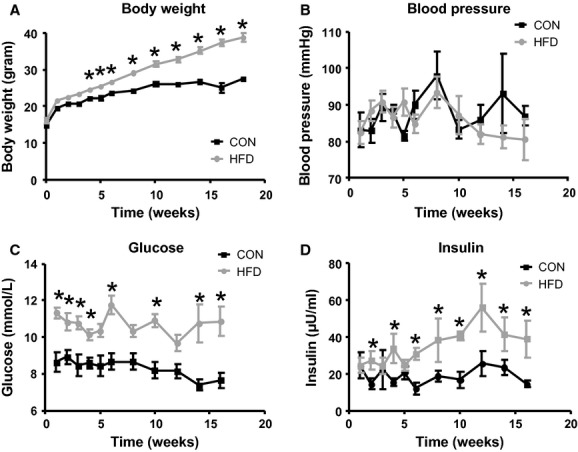
Animal characteristics. Weekly measurements of body weight (A), blood pressure by the tail cuff method (B), and blood glucose (C) and plasma insulin levels (D) via the saphenous vein were performed after a morning fast (4 h) in conscious mice that received chow (black line) or a HFD (gray line) for 6 and 18 weeks. As it was presumed that baseline glucose levels, that is, before the start of the diet, would not be different between animals, the first glucose measurements were performed 1 week after the start of the HFD diet or chow diet. The first values presented in Figure 1C are, therefore, the glucose values measured 1 week after the start of the HFD diet or chow diet. Body weights and insulin levels were significantly different between diets, as well as in time. The glucose levels were significantly different between diets as well, however, not in time. There were no significant differences in blood pressure. *P < 0.05 compared to control diet (Bonferroni post hoc test).

Body weight, blood pressure, baseline glucose, and insulin levels were measured after a morning (4 h) fasting period; the first measurements were performed 1 week after placement of the animals on their respective diet (chow or HFD). Body weight (Fig. 1A) and insulin levels (Fig. 1D) were significantly different between diets, as well as in time. Glucose levels were significantly different between both treatments as well, however, not in time (Fig. 1C). There were no significant differences in blood pressure (Fig. 1B).

### Glycocalyx barrier properties in muscle

**Figure 2.**
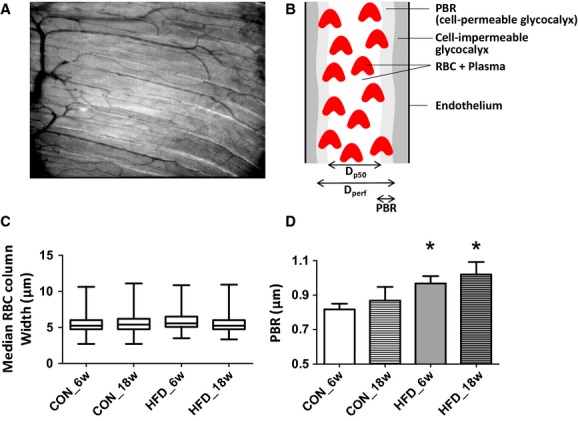
Hindlimb imaging. (A) Typical example of a single SDF image of the mouse hindlimb muscle microcirculation; full movie can be found in the Appendix. From each movie (total length: 100 frames) recorded by the SDF camera the distribution of the width of the red blood cell (RBC) column in each visible microvessel was determined (see Methods). (B) Cartoon depicting the median RBC column width, perfused diameter, and the PBR in a blood vessel. (C) Distribution of median RBC column width of all measured vessels segments (*n* = 1575 in CON_6w, *n* = 1378 in CON_18w, *n* = 1493 in HFD_6w, and *n* = 1391 in HFD_18w) in the hindlimb muscle of mice fed chow for 6 weeks (*n* = 7), a HFD for 6 weeks (*n* = 8), chow for 18 weeks (*n* = 7), and HFD for 18 weeks (*n* = 7). (D) Mean ± SEM of PBR measured in the vessel segments with a median RBC column between 4 and 6 *μ*m. *P < 0.05 compared to CON_6w (unpaired *t*‐test).

The PBR in the skeletal muscle microcirculation of the mice that received chow for 6 weeks was 0.81 ± 0.03 *μ*m, and this parameter was significantly increased in the mice that were fed a HFD during this period (0.97 ± 0.04 *μ*m) as well as in mice that were fed a HFD for 18 weeks (1.02 ± 0.07 *μ*m) (Fig. 2D). The PBR between the mice that received chow for 6 and 18 weeks (PBR in CON_18w was 0.87 ± 0.08 *μ*m), as well as between the mice that were fed a HFD for 6 and 18 weeks, was not significantly different.

### Intraperitoneal glucose tolerance test

An IPGTT was performed after an overnight fast under anesthesia after 6 weeks and 18 weeks of chow or HFD. Baseline glucose levels, as well as insulin levels, were not different between the mice that received chow for 6 and 18 weeks before the IPGTT (respectively, 6.1 ± 0.2 mmol/L and 11.9 ± 1.5 *μ*U/mL after 6 weeks vs. 6.0 ± 0.4 mmol/L and 11.0 ± 1.2 *μ*U/mL after 18 weeks). Furthermore, in contrast to the increased baseline blood glucose and plasma insulin levels in the HFD mice measured in the conscious state after a morning fast, glucose levels and insulin levels appeared not increased at baseline before the IPGTT at 6 weeks (6.9 ± 0.5 mmol/L and 13.3 ± 2.4 *μ*U/mL) when mice were under anesthesia. In the 18‐week HFD‐fed mice, baseline glucose levels were also not different (7.2 ± 0.5 mmol/L), whereas insulin levels tended to be increased (18.7 ± 2.4 *μ*U/mL; *P* = 0.08) in these mice.

**Figure 3.**
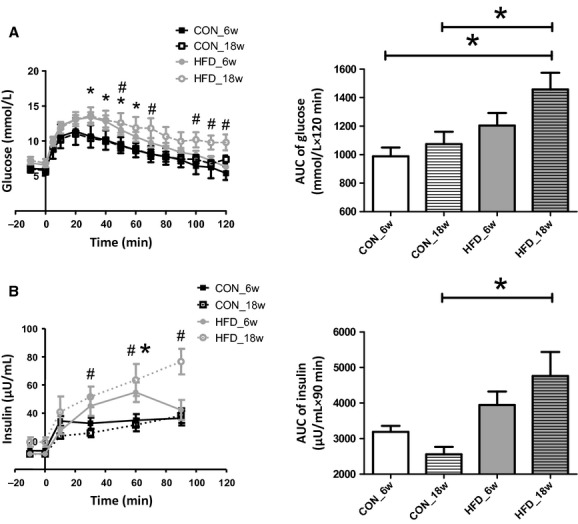
Glucose tolerance tests. (A) Mean ± SEM of blood glucose in time after an i.p. bolus of glucose (1 g/kg) (left panel). As a reflection of the glucose levels during the IPGTT the total area under the curve (AUC) was calculated for the period of 0–120 min after glucose infusion by the linear trapezoidal rule in mice fed chow for 6 weeks (*n* = 5), a HFD for 6 weeks (*n* = 7), chow for 18 weeks (*n* = 6), and HFD for 18 weeks (*n* = 8). *P < 0.05, HFD_6w compared to CON_6w (unpaired *t*‐test); ^#^*P* < 0.05, HFD_18w compared to CON_6w. (B) Mean ± SEM of plasma insulin levels in time after an i.p. bolus of glucose (1 g/kg) (left panel). As a reflection of the insulin levels at the end of the IPGTT, the total AUC of insulin levels was calculated for the period of 0–90 min after glucose infusion by the linear trapezoidal rule. *P < 0.05, HFD_6w compared to CON_6w (unpaired *t*‐test); ^#^*P* < 0.05, HFD_18w compared to CON_6w.

After i.p. injection of glucose in the 6 weeks chow mice, blood glucose levels increased to a peak of 11.4 ± 0.7 mmol/L after 20 min, and then gradually returned to baseline after 120 min (Fig. 3A). Plasma insulin levels were increased at the first measurement (10 min) and remained elevated for up to 90 min after the glucose injection (Fig. 3B). Glucose and insulin responses did not differ in the mice that were fed chow for 18 weeks. In the mice that received a HFD for 6 weeks, the peak in blood glucose was 13.1 ± 0.6 mmol/L and occurred after 30 min, while similarly returning to baseline after 120 min (Fig. 3A). As a result, the corresponding AUC of glucose was not increased after a HFD for 6 weeks during the IPGTT (*P* = 0.11). The initial insulin response after 10 min was comparable to that in chow animals; however, insulin levels further increased up to 60 min after glucose infusion (Fig. 3B), after which they decreased to a level comparable to that in chow mice after 90 min. After a HFD for 18 weeks, peak glucose showed a similar response as in the mice fed the HFD for 6 weeks, but the subsequent return to baseline was incomplete, resulting in increased glucose levels after 120 min. Consequently, the AUC of glucose during the IPGTT was significantly increased in these animals (*P* < 0.05; Fig. 3A). Furthermore, insulin levels increased gradually after the glucose administration, resulting in a significant increase in plasma insulin concentration at 30, 60, and 90 min. (*P* < 0.05; Fig. 3B).

### Relation between glycocalyx barrier properties and insulin‐mediated glucose disposal

**Figure 4.**
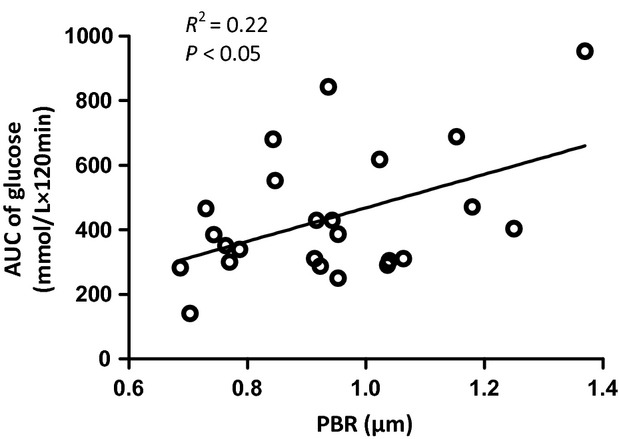
Relation between PBR and AUC of glucose. Relation between the PBR measured at baseline (*X*‐axis) and the corresponding AUC of glucose measured during the IPGTT (*Y*‐axis) for all individual experiments in this study in which both PBR and AUC glucose for 120 min were obtained. Correlation analysis revealed a *R*^2^ of 0.22 (*P* < 0.05).

As our previous data (Eskens et al. 2013) demonstrated that damage to the glycocalyx was associated with an impaired ability for insulin to dispose glucose, we related the PBR to the AUC of glucose during the IPGTT in all individual animals for the four groups (Fig. 4). There was a significant correlation between the baseline PBR and AUC of glucose measured during the IPGTT, suggesting that a greater reduction in glycocalyx barrier properties in muscle was associated with a greater inability to dispose the administered glucose bolus.

### Muscle capillary density

**Figure 5.**
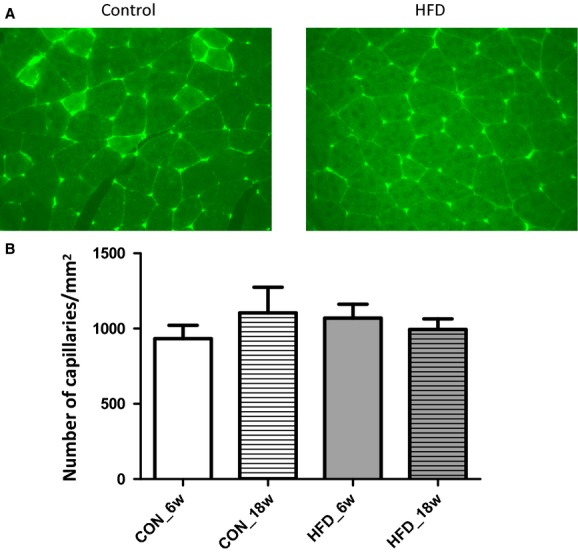
Structural capillary density. (A) Representative images of FITC‐WGA staining in hindlimb muscle of control mice (left image) and of HFD‐treated mice (right image). Intensity of staining appeared not different between control and HFD mice; also, the number of stained capillaries was comparable. (B) Average data of structural capillary density in hindlimb muscles of mice that were fed chow for 6 weeks (*n* = 4), chow for 18 weeks (*n* = 5), HFD for 6 weeks (*n* = 4), or HFD for 18 weeks (*n* = 5).

Typical examples of the FITC‐WGA staining of muscle are shown in Figure 5A. No differences in muscle capillary density were observed in the mice that were fed a HFD for 6 weeks or 18 weeks, or chow for 18 weeks, compared to the mice that received chow for 6 weeks (Fig. 5B).

## Discussion

In the microcirculation, the RBC column is separated from the endothelium by a gap at both sides (Vink and Duling 1996); Henry and Duling 1999); Broekhuizen et al. 2010). An important part of this cell‐free layer consists of the glycocalyx (Kim et al. 2009), a compartment that has been demonstrated to protect the endothelium against harmful stimuli (van den Berg et al. 2003); Reitsma et al. 2007) and to control tissue delivery of insulin (Eskens et al. 2013). Although it was previously shown that the dimensions of the glycocalyx were reduced in type II diabetes (Broekhuizen et al. 2010) and that severe hyperglycemic and hyperlipidemic conditions induce glycocalyx damage (Vink et al. 2000); Zuurbier et al. 2005); Nieuwdorp et al. 2006); van den Berg et al. 2009), there is little known about the presentation of glycocalyx loss in relation to the disturbed glucose homeostasis during obesity. In this study we assessed glycocalyx barrier properties in skeletal muscle using SDF imaging at an early and later stage of DIO in mice, by feeding them a HFD for 6 and 18 weeks, respectively. Glycocalyx barrier properties in hindlimb muscle microcirculation were found to be impaired after 6 weeks already. Our results suggest that in obesity glycocalyx damage represents an early aspect of microvascular dysfunction which may as well contribute to the development of glucose intolerance.

### Impaired glucose tolerance development in HFD‐fed mice

The HFD‐fed mouse model has been used as a robust model to study the development of impaired glucose tolerance and early type II diabetes, and resembles human obesity more closely than other (genetic) mouse models. In a previous study from our group it was shown as a proof of principle that placement of C57Bl/6 and ApoE3‐Leiden mice on a HFD for a period of 3 months was associated with loss of glycocalyx in capillaries as studied by intravital microscopy of the cremaster microcirculation (Constantinescu et al. 2011). By using a comparable dietary intervention we aimed in this study to examine an early occurrence of glycocalyx damage in hindlimb muscle, and relate this to the time of development of glucose intolerance. In the HFD‐fed mouse model, increases in fasting blood glucose levels and plasma insulin levels have been reported to appear already after 1 week of HFD, resulting in a stable hyperglycemia and a progressively increased hyperinsulinemia over time (Ahren and Pacini 2002); Winzell and Ahren 2004). These data indicate that in this model hepatic insulin sensitivity is rapidly deteriorated and partly compensated by an increased insulin release (Del Prato et al. 2002); Nathan et al. 2007). In line herewith, we observed increases in blood glucose after a morning fast in the conscious animals already after 1 week of HFD feeding which were more or less constant in the following weeks, whereas insulin levels more slowly increased over time, becoming significantly elevated after 6 weeks of diet (Fig. 1). In contrast to these measurements in the conscious state, baseline glucose levels were not significantly different between the control animals and HFD‐fed animals when put under anesthesia (Fig. 3A). This finding is in agreement with a previous study in mice, which used a similar type of anesthesia and HFD composition as we did in this study; no differences in baseline glucose levels were found after feeding a HFD for 1, 3, or 10 months in this study (Ahren and Pacini 2002). The authors of this study also reported increases in baseline insulin levels after feeding a HFD for 3 months. For comparison, in this study, baseline insulin levels tended to be increased (*P* = 0.08) after 18 weeks of HFD when the animals were under anesthesia. Besides the effect of anesthesia, the difference in duration of fasting between the conscious (4 h fast) versus anesthetized (overnight fast) mice may have contributed to the disparity in baseline glucose levels between the two experimental conditions. It has been shown that prolonged fasting impairs baseline insulin sensitivity in chow as well in HFD‐fed mice (Andrikopoulos et al. 2008). In agreement, although we found a significant difference in baseline glucose levels between the conscious chow and HFD mice, which had undergone only a 4 h fast, this difference was not present anymore after an overnight fast with the animals under anesthesia.

As a measurement for muscle insulin sensitivity (Nathan et al. 2007), we performed an IPGTT after 6 and 18 weeks of feeding a HFD (or chow) while the animal was under anesthesia. Although peak glucose levels were increased and delayed after 6 weeks of HFD, the recovery of blood glucose to baseline seemed not yet affected resulting in no significant increase for the AUC of blood glucose. In contrast, in the mice that received the HFD for 18 weeks the recovery of blood glucose was greatly impaired and glucose levels significantly elevated after 2 h (Fig. 3A), resulting in a significant increased AUC of glucose. Similarly, while insulin levels had returned to normal 90 min after the glucose infusion in the mice that received a HFD for 6 weeks, these were still significantly elevated in the 18 weeks of the HFD group (Fig. 3B). These data describe, in line with previous studies (Ahren and Pacini 2002); Winzell and Ahren 2004), the progressive decrease in muscle insulin sensitivity in mice upon feeding a HFD. The development of insulin resistance is accompanied by an increased insulin secretion, which initially seems to enable normal glucose tolerance to be maintained (Bergman et al. 2002), yet is insufficient to compensate for the decrease in insulin sensitivity after 18 weeks of HFD, as indicated by the hyperinsulinemia yet significant glucose intolerance.

### Glycocalyx barrier properties in HFD‐fed mice

DIO has been associated with endothelial dysfunction, mainly measured in aortic rings and mesenteric arteries (Molnar et al. 2005); Sachidanandam et al. 2009); Ketonen et al. 2010). However, insulin‐mediated glucose uptake occurs principally in skeletal muscle (Baron 1994), where the microcirculation regulates the delivery of insulin toward the myocytes (Baron 1994); Rattigan et al. 2013). The notion is that insulin, by stimulating the endothelium, increases blood volume in the muscle capillaries and thereby facilitates its own transport toward the myocytes (Vincent et al. 2004); Barrett et al. 2011). Consequently, an impaired microvascular insulin response due to endothelial dysfunction has been suggested to contribute to the development of insulin resistance (de Jongh et al. 2004); Clark 2008). Recently, we provided evidence that the glycocalyx plays a role in the insulin‐stimulated capillary blood volume increase and associated glucose disposal (Vlahu et al. 2012); Eskens et al. 2013). We, therefore, tested in this study if glycocalyx damage is relevant in the process of insulin resistance development during prodiabetic conditions. Glycocalyx barrier properties were measured in the hindlimb muscle capillaries of obese mice by using SDF imaging and analysis of the outward variations in RBC column width (Vlahu et al. 2012); Donati et al. 2013); Martens et al. 2013); Mulders et al. 2013). This clinically available methodology has recently been introduced and the reported data have shown impaired glycocalyx barrier properties in the sublingual microcirculation of dialysis patients (Vlahu et al. 2012), patients with premature coronary artery disease (Mulders et al. 2013), in lacunar stroke patients (Martens et al. 2013), and critically ill patients (Donati et al. 2013). In addition, we used the technique for monitoring glycocalyx barrier properties in the rat hindlimb muscle microcirculation in our previous study (Eskens et al. 2013). The analysis aims at an assessment of the temporal penetration depth of RBCs into the endothelial glycocalyx, which represents one aspect of the glycocalyx barrier. The rationale behind the measurement is that when the glycocalyx gets damaged, the barrier function becomes compromised enabling more RBCs to penetrate further into the glycocalyx, and this will be associated with an increase in PBR. To verify that damage to the glycocalyx is truly associated with an increase in PBR as a result of the outward radial displacement of circulating RBCs, we have recently performed additional intravital microscopy observations. For this experiment, mice (FVB/N background) which express a green fluorescent protein (GFP) under the direction of the endothelial‐specific receptor tyrosine (Tie2) were used (GFP‐EC mice). These GFP‐EC mice were prepared for intravital microscopic observation of the cremasteric microcirculation (*n* = 7) as described previously (VanTeeffelen et al. 2005); Constantinescu et al. 2011). Microvessels (*n* = 16 total) were alternately observed at high magnification using bright‐field microscopy for depiction of the RBC column and epi‐illumination for examination of the GFP signal using the appropriate filters for fluorescein. The anatomic vessel width was determined by the position of the endothelium by the GFP intensity peaks, whereas the perfused diameter was measured by determining the width of the RBC profile at half height intensity; from these measurements, the RBC‐EC gap, that is, the space between endothelial cells and RBC column, was determined by calculating the difference between the vessel diameter and the perfused diameter divided by 2 (as the gap is present on both sides of the RBC column). Paired measurements, performed before and 30 min after hyaluronidase treatment (35 U, jugular vein infusion) (van den Berg et al. 2003); Potter et al. 2009); VanTeeffelen et al. 2013), revealed that enzymatic degradation resulted in an outward movement of the outer edge of the RBC column toward the endothelium by 0.8 ± 0.3 *μ*m, without a significant change in vessel diameter (data not shown). These data indicate that increases in PBR are indeed indicative of an impaired glycocalyx barrier.

In this study, glycocalyx barrier properties were measured in capillaries with a median RBC column width between the 4 and 6 *μ*m, which coincided with a PBR of 0.81 ± 0.03 *μ*m in control mice. Feeding a HFD for 6 weeks caused the PBR to be significantly increased with 0.15 ± 0.04 *μ*m. In the following 12 weeks of the diet, the PBR remained elevated at this level while intolerance to glucose infusion clearly developed. The magnitude of increase in PBR as a result of a compromised glycocalyx barrier is in line with a previous intravital microscopic study in mice fed a HFD for 3 months, which showed a 0.2–0.3 *μ*m reduction in glycocalyx thickness in cremaster muscle capillaries (Constantinescu et al. 2011). The data of this study, therefore, suggest that glycocalyx damage was manifested already after 6 weeks of HFD and, based on our previous study (Eskens et al. 2013), it is anticipated that this impairment may have contributed to the worsening of glucose tolerance.

Although a decrease in insulin sensitivity during obesity has been related to a decrease in the number of capillaries present (Baron 1994); de Jongh et al. 2004); Clark 2008), we did not observe significant changes in capillary density in the hindlimb muscle of the mice that were fed a HFD for 6 or 18 weeks (Fig. 5). We used lectin staining to measure capillary density, which has been commonly used for evaluation of structural capillary density (Van Kerckhoven et al. 2004); Frisbee 2005). Although WGA has an affinity for carbohydrate moieties present in the glycocalyx (N‐acetylneuraminic acids and N‐acetyl‐*β*‐D‐glucosamine), the staining appeared not to be affected by the HFD‐associated glycocalyx breakdown. This is likely explained by the fact that the carbohydrate residues to which WGA can bind are not exclusive for the endothelial glycocalyx but may include abluminal capillary structures such as the basement membrane as well. In agreement, in a recent study in mice, WGA staining of the glomerulus was found not to be affected by hyaluronidase treatment, in contrast to two other lectins (LEA and BSI) (Dane et al. 2013). Rather than structural changes in the microcirculation of the muscle, it is suggested, therefore, that the decrease in insulin sensitivity in these mice may have resulted from an impaired insulin delivery to the myocytes due to an impaired ability to recruit capillary blood volume or to transport insulin to and across the endothelium because of the affected glycocalyx (Eskens et al. 2013). While 6 weeks was chosen as the earliest time point for assessment of glycocalyx loss during the development of DIO in this study, earlier changes in glucose metabolism after start of the HFD, such as those reflected by the increase in fasting plasma glucose levels after 1 week already (Fig. 1C), could as well have initiated glycocalyx damage earlier in the development of DIO. Short‐term hyperglycemia in itself, albeit at much higher levels than occurring in this study, was previously shown to diminish glycocalyx barrier properties (Zuurbier et al. 2005). In line herewith, St‐Pierre et al. (2010) showed in a recent study that the ability of insulin to recruit microvascular blood volume in skeletal muscle in a rat model was already impaired after feeding a HFD for 4 weeks, and this impaired microvascular response was associated with an impaired insulin‐mediated glucose uptake during an isoglycemic hyperinsulinemic clamp. In summary, the data of this study indicate early damage to the glycocalyx, before the development of overt glucose intolerance, in a mouse model of DIO. These findings indicate that glycocalyx damage may well underlie the reported association between endothelial dysfunction and impaired insulin action during obesity. Future studies will be needed, however, to resolve the true staging of glycocalyx damage development during DIO, and to substantiate the contribution of this damage to the development of insulin resistance and glucose intolerance.

## Conflict of Interest

None declared.
